# Supplementary material for: Phenotypic and Genotypic Adaptation of Escherichia coli to Thermal Stress is Contingent on Genetic Background
Source: Mol Biol Evol. 2023 May 4;40(5):msad108. doi: 10.1093/molbev/msad108 (PMC10195153; doi:10.1093/molbev/msad108)
Supplement: msad108_Supplementary_Data [file msad108_supplementary_data.zip › 2023-04-04_SuppFigures.docx]

**Supplementary Figures**

**Supplemental Figure 1:** Fitness values for a subset of candidate Phase 1 evolved lines from Tenaillon et al. (2012) carrying one mutation in either *rho* (dots) or *rpoB* (triangles). The fitness measures were taken for two temperatures: 19.0°C and 42.2°C. This data was part of the criteria used to select the Phase 1 evolved populations to serve as founders for Phase 2. Fitness values and was previously measured and reported in Tenaillon et al. (2012) and Rodríguez-Verdugo et al. (2014). Evolved lines that were selected to serve as Phase 2 Founders are colored in blue

**Supplemental Figure 2:** All detected mutations at a frequency of 5% or higher in the Phase 2 evolved populations represented along the *E. coli* chromosome. Mutational types are distinguished by the shape of the point and the intensity of the color corresponds to the frequency that mutation was found in the population. Populations are grouped to the right according to their Phase 2 Founder historical background.

**Supplemental Figure 3:** Maximum likelihood phylogeny built from all mutation data across both phases of the experiment. Sequencing data from the ancestral *E. coli* REL606, the Phase 1 Founders, and the Phase 2 evolved populations were used to build the phylogeny. Branches are colored according to the Phase 2 Founder historical background: *rpoB* = red, *rho* = blue, and ancestral (REL1206, Phase 1 Ancestor) = gold.

**Supplemental Figure 4:** Dice’s similarity coefficients calculated from total (5% or higher frequency) mutation data, separated by the type of pairwise comparison
